# Historical account of reperfusion therapy and the development of cardioprotection beyond reperfusion

**DOI:** 10.1007/s00395-026-01198-1

**Published:** 2026-07-17

**Authors:** Gerd Heusch, Petra Kleinbongard, Bernard J. Gersh

**Affiliations:** 1 Cardioprotection Unit, Institute for Pathophysiology, West German Heart and Vascular Center, University of Duisburg-Essen, Hufelandstr. 55, 45147 Essen, Germany; 2 Department of Cardiovascular Medicine, Mayo Clinic College of Medicine and Science, Rochester, MN USA

**Keywords:** Cardioprotection, Myocardial infarction, Percutaneous coronary intervention, Reperfusion, Thrombolysis

## Abstract

The only way to salvage myocardium from infarction is timely reperfusion of the occluded coronary artery. Timely reperfusion reduces infarct size and mortality. The history of reperfusion therapy from the first clinical use of intravenous streptokinase in patients with acute myocardial infarction by Sherry et al. in the late 1950s, over seminal experimental dog studies by Ross Jr. et al., demonstrating infarct size reduction by timely reperfusion in the early 1970s, to a more systematic use of initially streptokinase and a later tissue plasminogen activator in clinical trials (GISSI, TIMI), the addition of platelet inhibition (ISIS) and ultimately mechanical recanalization by percutaneous coronary intervention with stenting, is reviewed in detail. Cardioprotection beyond timely reperfusion was first demonstrated in the seminal experimental dog studies of ischemic conditioning by Murry, Reimer, and Jennings in the 1980s and initiated a tsunami of experimental studies in various experimental models to identify the underlying signal transduction, but has not yet been established in clinical practice. Clinical trials on various forms of ischemic conditioning, potentially cardioprotective drugs and transcutaneous vagal nerve stimulation, are still underway.

## Introduction

Until 4–5 decades ago, acute myocardial infarction (AMI) resulted in a high in-hospital mortality of about 20% in clinical trials, not even accounting for the mortality of those who never reached the hospital and those who died after discharge from the hospital. Reperfusion therapy has since revolutionized the management of ST segment elevation myocardial infarction (STEMI) with reduction in-hospital mortality to about 5% or less [3, 36, 42], and the presence of an open artery post-reperfusion together with newer pharmacological approaches has improved the late prognosis of survivors [4]. There are many unjustified claims who deserves credit for inventing reperfusion therapy of AMI. In fact, reperfusion therapy roots in several important observations, experimental studies, and clinical trials as well as their mutual interaction. It appears that the early clinical studies on the use of streptokinase in patients with acute myocardial infarction by Sherry in the late 50s [16, 17] were not followed up and often not even credited retrospectively. After the seminal experimental dog studies by Maroko, Ross and Braunwald in 1971 and 1972 demonstrated reduced infarct size by hemodynamic interventions [63], of note by reperfusion even after 3 h coronary occlusion [20], and after Reimer and Jennings had demonstrated in dog experiments with coronary occlusion of different durations before reperfusion the wavefront expansion of myocardial infarction [72], reperfusion therapy had just become an idea whose time had come. We now have the answers to many questions in the field, and the most important issues we face are logistical—the achievement of prompt reperfusion therapy in everyone.

### Early clinical use of intravenous thrombolysis

Herrick had already suggested that AMI in patients was caused by coronary obstruction with a thrombus [26], and this suggestion was many years later confirmed by DeWood et al. using coronary angiography [12]. Sherry and coworkers were the first to consequently use thrombolysis with intravenous streptokinase in a small cohort of patients with AMI, and they reported improved clinical outcomes and reduced mortality when treatment was started “early” (9–16 h after symptom onset) [16, 17]. Despite these favorable results, intravenous thrombolysis for treatment of AMI was not widely adopted for the following 2 decades.

### Experimental recognition of reperfusion to reduce infarct size

It was not until 1972 that reperfusion after 3 h coronary occlusion in conscious dogs was proven to reduce infarct size in studies with John Ross Jr. as a senior author [20]. Maroko, Ross, Braunwald and co-workers in the same institution had just reported one year earlier that infarct size could be modified by various hemodynamic interventions during early coronary occlusion, but that study had surprisingly not included reperfusion [63]. The “wavefront” concept of lateral and transmural spreading of infarction over the duration of coronary occlusion in dogs was developed by Reimer and Jennings several years later [72]. After these earlier studies, infarct size has become and remained the gold standard of all preclinical studies on reperfusion therapy and cardioprotection [1, 56].

### Intracoronary thrombolysis and mechanical coronary recanalization

Intravenous urokinase in combination with heparin reduced ECG signs of infarction and mortality at 30 days in a small patient cohort in 1975 [5], but thrombolysis with urokinase was never followed up in larger clinical trials [91]. In 1976, Chazov in Moscow published favorable results of intracoronary streptokinase in patients with AMI [8], and while this study went unnoticed in the West, Rentrop and coworkers in Göttingen successfully demonstrated the efficacy of intracoronary streptokinase in patients with AMI in 1979 [73]. Incidentally, Rentrop et al., one year earlier, were the first to report mechanical recanalization by a guide wire in a patient with AMI [74, 75], before a few years later Meyer et al. [65] and shortly thereafter in a larger patient cohort, Hartzler et al. reported percutaneous transluminal angioplasty with and without thrombolysis [23].

### Firm evidence for timely reperfusion from clinical trials

After the initial reports of successful thrombolysis and mechanical recanalization, reperfusion therapy gained momentum, and thrombolysis initially dominated. Intracoronary thrombolysis was logistically not feasible for a treatment early in the course of AMI in many patients [91], and the first large clinical trial of reperfusion therapy(GISSI trial) was in 1986 which used intravenous streptokinase in patients admitted within 12 h of symptom onset and provided unequivocal evidence for reduced in-hospital mortality [21]. Only a year later, the first thrombolysis in myocardial infarction (TIMI) trial achieved a higher reperfusion rate with recombinant tissue plasminogen activator (tPA) [90]. In this trial also the TIMI flow grade to judge the success of reperfusion angiographically was developed [84]. The International Study of Infarct Survival (ISIS)-2 trial in 1988 provided evidence that additional platelet inhibition by oral aspirin provided additional benefit beyond streptokinase alone in all-cause mortality over 15 months follow-up [45]. The Global Utilization of Streptokinase and Tissue Plasminogen Activator for occluded coronary arteries (GUSTO) trial in 1993 confirmed the superiority of tPA in conjunction with heparin over streptokinase with heparin in terms of vessel patency and reduction of early (30 days) mortality [22]. In patients with traditional contraindications for thrombolysis, reperfusion with primary angioplasty reduced in-hospital and 6-months mortality more than tPA [87]. Immediate mechanical recanalization resulted in greater myocardial salvage than thrombolytic therapy in the Mayo Coronary Care Unit and Catheterization Laboratory Groups [19]. Stenting reduced restenosis better than balloon angioplasty [62], and direct stenting without prior balloon dilatation resulted in less coronary microvascular injury [60], possibly by rapidly sealing the ruptured plaque and attenuating coronary microembolization [14, 50]. Primary angioplasty with or without stenting was found to reduce all-cause mortality more than thrombolysis in a meta-analysis of 23 trials in 2003, establishing mechanical recanalization by primary percutaneous coronary intervention (PCI) as the preferential method of reperfusion [47]. The TIMI program which Braunwald and coworkers had initially used to study the superiority of tPA over streptokinase has been developed by them over the following decades into an excellent and highly professional infrastructure for randomized clinical trials which further refined the agents, procedures, and algorithms of reperfusion therapy It was also extended to use in other cardiovascular therapy trials. The current best practice of reperfusion according to ESC guidelines [7] in patients with STEMI is reperfusion therapy in patients who present within 12 h of symptom onset, a primary invasive approach of mechanical recanalization when they present within 120 min of symptom onset and a thrombolytic approach if primary PCI is not possible within 120 min of symptom onset, always in conjunction with platelet inhibition. Whereas reperfusion should be initiated as early as possible after symptom onset and to be followed by angiography before hospital discharge, salvage of myocardium was seen as reduced infarct size on scintigraphy when patients with AMI who presented after 12–48 h of symptom onset and underwent stenting and platelet inhibition by abciximab [80]. The benefit over conservative treatment persisted in mortality over 4 years of follow-up [67]. The  group of Schömig and co-workers attributed such late benefit from reperfusion to a short-term hibernation process of self-protection in the ischemic myocardium, as initially proposed by Rahimtoola for self-protection in ischemic cardiomyopathy, which was reversible by revascularization in 1985 [71] and later identified in mechanistic animal studies [32, 64, 78, 82]. In line with preclinical studies, infarct size was also identified as the most important determinant of patients´ prognosis after primary PCI [88].

### Reperfusion injury

Timely reperfusion not only salvages myocardium from infarction but also comes at a price. Interestingly, injury to the reperfused coronary circulation was first recognized as there was no-reflow following release of a prolonged coronary occlusion in animal studies [52, 54]. The mechanisms underlying coronary microvascular injury during reperfusion [10, 31, 50] and their clinical importance for patient outcome [11] were only recognized many years later [30, 68]. Prolonged contractile dysfunction after the release of a coronary occlusion of too short duration to cause irreversible injury (stunning) was first recognized in dog experiments by Heyndrickx, Vatner, and coworkers in 1975 [32, 41]. Whether or not reperfusion also caused irreversible injury beyond that by the preceding ischemia or just facilitated its morphological detection was long debated [40]. Yellon and coworkers in 2002 first demonstrated in isolated rat hearts that an intervention at reperfusion (urocortin) could reduce infarct size, thus providing evidence that irreversible injury was ongoing during reperfusion but could be prevented [81]. Final proof for irreversible injury to cardiomyocytes was only provided when Zhao, Vinten-Johansen, and coworkers in 2003 demonstrated in dogs that infarct size could be reduced by repetitive brief coronary occlusion/reperfusion at early reperfusion (ischemic post-conditioning) [94], emphasizing that infarct size still increases during early reperfusion and can still then be reduced by an intervention.

### Cardioprotection only in conjunction with reperfusion

The initial studies to limit infarct size by hemodynamic or pharmacological interventions during coronary occlusion in the absence of reperfusion were not reproduced with a more advanced and rigorous technology (triphenyl tetrazolium chloride staining, normalization to area at risk and residual blood flow) [1]. The initial enthusiasm about infarct size limitation waned subsequently. Cardioprotection could only be developed after the recognition of myocardial salvage from infarction by reperfusion, and any cardioprotective intervention has to prove its efficacy beyond that by reperfusion alone [35]. The idea of infarct size limitation was revived when Murry, Reimer, and Jennings in 1986 first reported ischemic preconditioning in dogs with infarct size reduction by 4 cycles of 5 min coronary occlusion/5 min reperfusion preceding 40 min coronary occlusion with reperfusion beyond that by reperfusion per se. This protection was only effective when reperfusion occurred earlier than after 3 h coronary occlusion [66]. Ischemic preconditioning can only be used clinically in elective situations but not in AMI. In contrast, ischemic post-conditioning by cycles of brief coronary occlusion and reperfusion following rather than preceding prolonged coronary occlusion reduced not only infarct size in dogs as first reported by Zhao, Vinten-Johansen, and coworkers [94], but was quickly translated to patients also with AMI, as first reported by Staat, Ovize, and coworkers [86]. Of particular interest for cardioprotection is the fact that infarct size reduction in the heart can be achieved by subjecting tissues or organs remote from the infarcting myocardium to cycles of brief ischemia and reperfusion, as first reported by Przyklenk, Kloner, and coworkers in 1993 [70]. This sort of cardioprotection by remote ischemic conditioning can also be recruited during ongoing coronary occlusion (perconditioning) not only in pigs [79] but also in patients before undergoing reperfusion for AMI, as first reported by Botker and coworkers [2, 34, 37]. These studies on ischemic conditioning have raised a tsunami of preclinical studies attempting to identify its mechanisms and signal transduction, and indeed many signaling steps of ischemic conditioning have been identified [27, 31]. Downey and coworkers first identified adenosine and activation of protein kinase C as preconditioning signals [59, 92]. Such studies were then extended to pathways such as the reperfusion injury salvage kinase (RISK) and the survivor activating factor enhancement (SAFE) pathways by Hausenloy and Yellon [25] and by Lecour [55]. Unfortunately, none of the many identified signals has so far materialized into a practical therapy. In parallel to the preclinical studies, a number of clinical studies have attempted to translate ischemic conditioning studies to patients with AMI—some with infarct size reduction or myocardial salvage [2, 13] or with better clinical outcome [18], and others without success [24]. The use of ischemic conditioning in patients with AMI has remained contentious and still requires final evidence [31, 36, 38]. Several molecular signals that had been identified in mechanistic preclinical studies were also targeted in clinical studies, again with neutral or contentious results e.g., for adenosine [6], inhibition of the mitochondrial permeability transition pore by cyclosporine [9, 69] or protein kinase C delta inhibition [58].

Notwithstanding the mass of strong experimental evidence and some positive signals in smaller clinical trials, cardioprotection has so far disappointingly failed to be translated into the clinical arena. A number of potential explanations have been identified [28], such as the use of young and healthy animals in preclinical mechanistic studies devoid of advanced age, comorbidities, and comedications, which are often present in patients with AMI [15, 49]. On the other hand, the excellent outcomes in contemporary studies with prompt and early reperfusion alone may by being a “victim of their own success” limit the prognostic benefit of additional myocardial salvage with cardioprotective interventions [36]. Ongoing trials therefore attempt to achieve cardioprotection by remote ischemic conditioning in patients who suffer from more severe hemodynamic impairment and higher Killip classes (NCT04844931) or who are underserved for logistic reasons [61]. Conceptually, it still remains unclear whether ischemic conditioning only limits reperfusion injury as suggested by the equal magnitude of protection by ischemic pre- and post-conditioning in the original study by Zhao et al. in dogs [94], or whether there is also a limitation of ischemic injury, as suggested by another study in pigs where ischemic preconditioning provided greater protection than ischemic post-conditioning [85] or a study with attenuation of ischemic ECG alterations during coronary occlusion by remote ischemic perconditioning [48].

Braunwald and coworkers had advocated the use of beta-blockade because it reduced oxygen consumption and reduced infarct size in their dog studies [63] and subsequent animal studies [44, 51, 83]. However, in the first clinical trial initiated and led by Braunwald, intravenous propranolol in patients, when given later than within 4 h of symptom onset, reduced heart rate, but not infarct size and mortality [76]. The use of beta-blockade for cardioprotection has remained contentious since then [43, 77], also questioning the supply–demand paradigm of Braunwald and its use for understanding cardioprotection, but emphasizing the need for rapid restoration of coronary blood flow by reperfusion [33]. Left ventricular unloading instead of immediate reperfusion reduced infarct size in pigs [89], but it remained unclear to what extent this effect was due to some reperfusion by improved collateral flow [39]. However, in patients with AMI, transvalvular left ventricular unloading failed to reduce infarct size by MRI and even caused more bleeding and vascular complications [46]. More recently, transcutaneous auricular vagus stimulation has been advocated for cardioprotection beyond reperfusion [29, 57], but this approach still requires more thorough clinical validation [53, 93].

**Fig.1:**
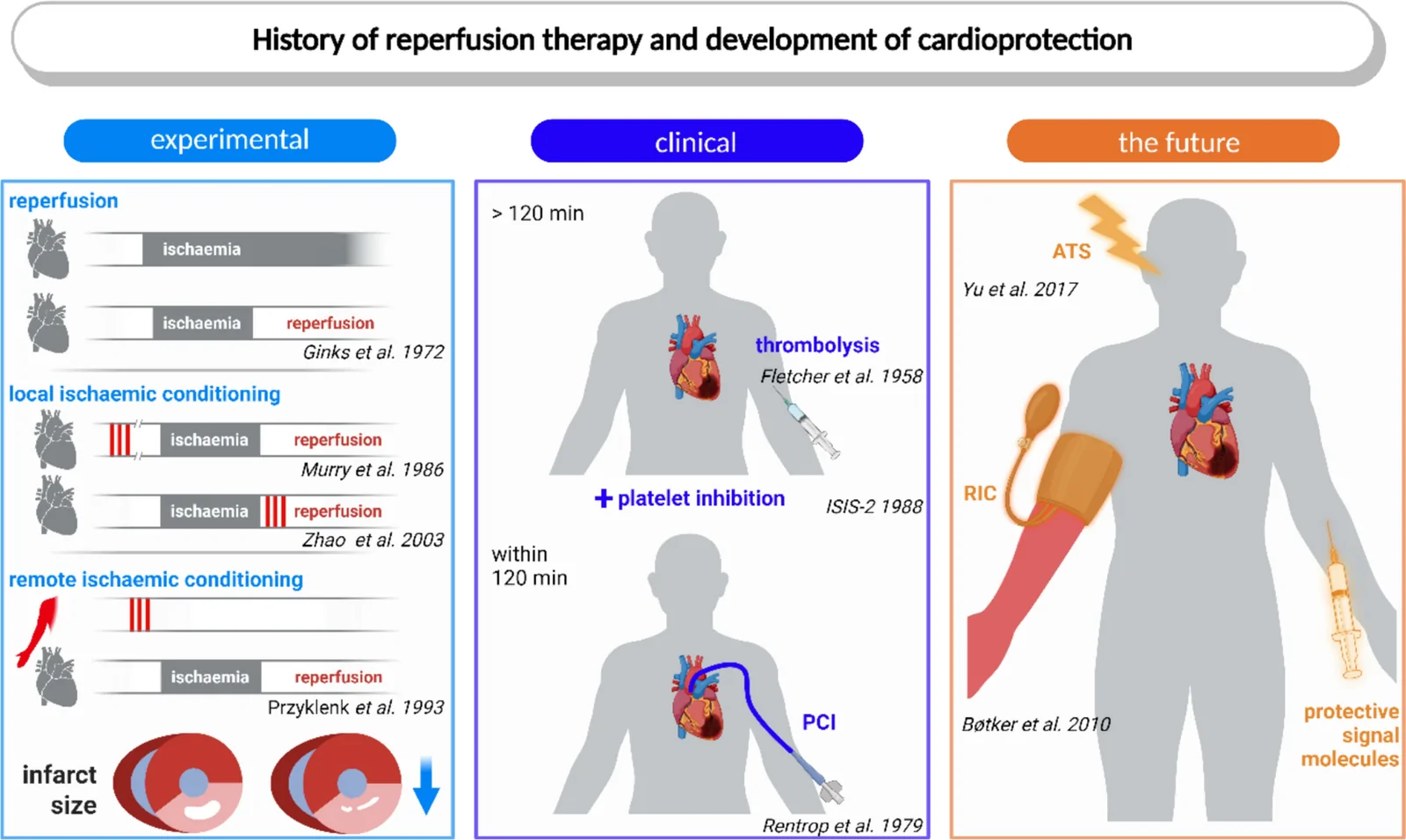
History of reperfusion therapy and development of cardioprotection in experimental and clinical studies, with a perspective to the future

In conclusion (Fig. 1), reperfusion therapy for AMI has made remarkable progress over the last decades and saved millions of lives. Many people have contributed to this success story. Cardioprotection beyond reperfusion has been difficult to translate into clinical practice so far, but nonetheless, it is still worth trying, particularly in patients at higher risks and those in many parts of the world who have difficulty in accessing to rapid reperfusion therapy.
